# Strengths and Weaknesses of ChatGPT Models for Scientific Writing About Medical Vitamin B12: Mixed Methods Study

**DOI:** 10.2196/49459

**Published:** 2023-11-10

**Authors:** Omar Abuyaman

**Affiliations:** 1 Department of Medical Laboratory Sciences Faculty of Applied Medical Sciences The Hashemite University Zarqa, 13133 Jordan

**Keywords:** AI, ChatGPT, GPT-4, GPT-3.5, vitamin B12, artificial intelligence, language editing, wide range information, AI solutions, scientific content

## Abstract

**Background:**

ChatGPT is a large language model developed by OpenAI designed to generate human-like responses to prompts.

**Objective:**

This study aims to evaluate the ability of GPT-4 to generate scientific content and assist in scientific writing using medical vitamin B12 as the topic. Furthermore, the study will compare the performance of GPT-4 to its predecessor, GPT-3.5.

**Methods:**

The study examined responses from GPT-4 and GPT-3.5 to vitamin B12–related prompts, focusing on their quality and characteristics and comparing them to established scientific literature.

**Results:**

The results indicated that GPT-4 can potentially streamline scientific writing through its ability to edit language and write abstracts, keywords, and abbreviation lists. However, significant limitations of ChatGPT were revealed, including its inability to identify and address bias, inability to include recent information, lack of transparency, and inclusion of inaccurate information. Additionally, it cannot check for plagiarism or provide proper references. The accuracy of GPT-4’s answers was found to be superior to GPT-3.5.

**Conclusions:**

ChatGPT can be considered a helpful assistant in the writing process but not a replacement for a scientist’s expertise. Researchers must remain aware of its limitations and use it appropriately. The improvements in consecutive ChatGPT versions suggest the possibility of overcoming some present limitations in the near future.

## Introduction

Artificial intelligence (AI) is a convergence of computer science and linguistics aiming to develop machines that emulate tasks necessitating human intelligence, including learning, adaptation, reasoning, comprehension, abstraction, and responsiveness to attributes like attention and emotion [1,2].The field of natural language processing has witnessed notable advancements in recent times, and one of the latest breakthroughs is the ChatGPT language model developed by OpenAI [3]. ChatGPT is an AI-based chatbot system. The ChatGPT model is a versatile general language model trained on a vast amount of text data, enabling it to generate human-like text responses based on a prompt. The launch of ChatGPT in late November 2022 (GPT-3.5 model) achieved the status of being the fastest-growing application in history by reaching 100 million users in under 2 months [4]. The recent advancements in ChatGPT have garnered significant attention within the scientific community. This is substantiated by the extensive volume of publications pertaining to ChatGPT indexed in PubMed, amounting to over 1000 articles as of August 2023. Concurrently, there is an observable escalation in competitive innovations in this field. Numerous forthcoming platforms, such as Google Bard, Microsoft Bing AI, Ernie bot, SearchGPT, and YaLM 2.0, among others, are on the cusp of introduction. While its primary use is in chatbots, ChatGPT’s versatility has led to its application in various fields, including potential use in scientific writing [5-10]. This has raised concerns among journals about how to use or limit the use of this new tool [11-18]. Scientific writing is a specialized form of writing that demands an in-depth understanding of the subject matter and mastery of the scientific language, making it a challenging task for even experienced researchers. ChatGPT has the potential to simplify the scientific writing process, saving time and effort for researchers to focus on other aspects of their work, such as research design and data analysis. Additionally, it could theoretically reduce errors and inconsistencies that are often found in scientific writing. A recent release of the ChatGPT model, GPT-4, boasts enhanced accuracy and heightened attention to details in user prompts, as demonstrated by OpenAI’s internal test results on an array of professional and academic exams [19]. However, its performance in the realm of scientific writing remains untested, and the results of OpenAI’s evaluations have not been verified through peer-reviewed publications. In this study, an evaluation was performed on GPT-4’s ability to generate scientific text on the specific topic of vitamin B12 treatment. In addition, the performance of GPT-4 was compared to that of its predecessor, GPT-3.5.

## Methods

### Study Design

The topic of medical vitamin B12 was selected due to the author’s prior expertise in the field and the belief that this area contains suitable issues for generating prompts that can effectively assess ChatGPT’s capabilities. The ChatGPT platform was accessed via the web-based interface [20], which is currently available at no cost for the GPT-3.5 model and for a subscription fee for the newly released GPT-4 model. ChatGPT’s responses were obtained through prompts, as detailed in the Results section, aiming to showcase the system’s workflow, strengths, and limitations. The generated text was received within seconds. Prompts submitted to ChatGPT ended with “Answer in one sentence”, though this phrase is not displayed herein. Each prompt was used twice, once for the GPT-4 model and once for the GPT-3.5 model. If similar answers were produced, only the GPT-4 response is presented herein. ChatGPT is aware of previous prompts on the same page, so to minimize variables and improve the repeatability of the answers featured in this article, each prompt was asked on a new ChatGPT page. Subsequently, the text generated by ChatGPT was assessed, primarily based on published articles in the PubMed database. Before finalizing the paper, each section was entered into GPT-4 for language refinement.

### Ethical Considerations

Given that the study did not engage with human participants or utilize personal data, ethical considerations are not applicable.

## Results

The results are categorized into 2 textboxes. The conversations presented in Textbox 1 served to evaluate the scientific text generated by ChatGPT, and those presented in Textbox 2 aimed to assess the editorial functions of ChatGPT. Each section is structured by asking ChatGPT to describe its capability followed by prompts that will test its performance. Prompts and answers by GPT-4 and GPT-3.5 are indicated, and a discussion of the results is given in the Discussion section.

ChatGPT models operate as a black box, meaning that the process by which it arrives at its answers is unknown. However, when prompted to describe the steps it uses to generate answers related to vitamin B12, its response was the following:

ChatGPT generates answers related to vitamin B12 by analyzing the user's query, accessing its vast training data, and synthesizing relevant information. It identifies important keywords, such as “vitamin B12,” connects them to related concepts, evaluates potential responses, and selects the most coherent and informative answer to present to the user.

**Evaluation of scientific text generated by ChatGPT. Prompts are indicated, followed by the responses by GPT-4 and GPT-3.5.**

**Text generation with proper references**

- *(Prompt) Can ChatGPT generate a scientific text?*
*(GPT-4) Yes, ChatGPT can generate scientific text by synthesizing relevant information and presenting it in a coherent and accurate manner within the scope of its training data.*
- *(Prompt) Does ChatGPT trained on published articles from 2022 and 2023?*
*(GPT-4) ChatGPT is trained on data up to September 2021 and does not include published articles from 2022 and 2023.*
- *(Prompt) Can ChatGPT add within text references from PubMed to its answers?*
*(GPT-4) ChatGPT cannot directly add within-text references from PubMed to its answers, as it is an AI language model and cannot access external databases in real-time.*
*(GPT-3.5) Yes, ChatGPT can add within text references from PubMed to its answers.*
- *(Prompt) What is vitamin B12? Add one reference from PubMed, keep the same text.*
*(GPT-4) Vitamin B12, also known as cobalamin, is an essential water-soluble nutrient involved in nerve function, red blood cell formation, and DNA synthesis (Green, R., & Allen, L. H. (2017). Vitamin B12 deficiency from the perspective of a practicing hematologist. Blood, 129(19), 2603-2611).*
*(GPT-3.5) Vitamin B12 is a water-soluble vitamin that is essential for various bodily functions, including red blood cell formation, DNA synthesis, and neurological function (O'Leary et al., 2018). Reference: O'Leary, F., Samman, S. (2018). Vitamin B12 in Health and Disease. Nutrients, 10(12), 1-20. doi: 10.3390/nu10121936.*

**Text accuracy and self-contradiction**

- *(Prompt) Are ChatGPT's responses regarding published scientific findings accurate?*
*(GPT-4) ChatGPT's responses regarding published scientific findings are generally accurate but may not always be up-to-date or comprehensive due to the knowledge cutoff and potential biases in training data.*
- *(Prompt) Do ChatGPT's responses contradict one another?*
*(GPT-4) ChatGPT's responses may sometimes contradict one another due to its training data and the inherent complexity of natural language understanding.*
*(GPT-3.5) It is unlikely that ChatGPT's responses would contradict one another, as it is designed to provide consistent and coherent answers based on the inputs and information available to it.*
- *(Prompt) Who needs treatment with vitamin B12?*
*(GPT-4) Individuals with vitamin B12 deficiency, often caused by pernicious anemia, malabsorption issues, strict vegetarian or vegan diets, or certain medications, may require vitamin B12 treatment.*
*(GPT-3.5) Individuals with vitamin B12 deficiency or pernicious anemia, as well as those with certain medical conditions or dietary restrictions, may need treatment with vitamin B12 supplements.*
- *(Prompt) How to treat pernicious anemia?*
*(GPT-4) To treat pernicious anemia, regular injections or high oral doses of vitamin B12 are administered, as the body cannot absorb this essential nutrient properly due to a lack of intrinsic factor.*
- *(Prompt) What is intrinsic factor? Can intrinsic factor be administered as an injection to treat Vitamin B12 deficiency?*
*(GPT-4) Intrinsic factor is a glycoprotein produced by the stomach's parietal cells, which is essential for the absorption of vitamin B12; it can't be administered as an injection, but vitamin B12 injections can be given to bypass the need for intrinsic factor and treat deficiency.*
*(GPT-3.5) Intrinsic factor is a glycoprotein produced in the stomach that facilitates the absorption of vitamin B12, and yes, it can be administered as an injection to treat vitamin B12 deficiency.*

**Bias and prioritizing**

- *(Prompt) Is it possible for ChatGPT to generate biased responses regarding scientific data?*
*(GPT-4) Yes, ChatGPT can potentially generate biased responses regarding scientific data due to its training on diverse and potentially biased human-generated text.*
- *(Prompt) Is cyanocobalamin the most common and effective form for vitamin B12 deficiency treatment?*
*(GPT-4) Cyanocobalamin is the most common form of vitamin B12 used for deficiency treatment, but methylcobalamin and adenosylcobalamin are considered more bioavailable and effective forms.*
*(GPT-3.5) Cyanocobalamin is the most commonly used and effective form of treatment for vitamin B12 deficiency.*

**Repeatability and reproducibility**

- *(Prompt) Are ChatGPT answers repeatable and reproducible?*
*(GPT-4) ChatGPT answers may not be entirely repeatable and reproducible, as the model can generate varying responses based on subtle differences in input or inherent randomness in its output.*
*(GPT-3.5) Yes, ChatGPT answers are repeatable and reproducible given the same input and context.*
- *(Prompt) What is the best way to treat vitamin B12 deficiency?*
*(GPT-4) The best way to treat vitamin B12 deficiency is through dietary changes, supplementation, or injections, depending on the severity and underlying cause of the deficiency.*
*(GPT-3.5) The most effective way to treat vitamin B12 deficiency is through regular injections or high-dose oral supplements of vitamin B12.*
- *(Prompt) What is the best way to treat vitamin B12 deficiency?*
*(GPT-4) The best way to treat vitamin B12 deficiency is through supplementation with oral or intramuscular B12, or by increasing the consumption of B12-rich foods, depending on the severity and underlying cause of the deficiency.*
*(GPT-3.5) The optimal treatment for vitamin B12 deficiency involves a combination of injections or high-dose supplements of vitamin B12 and addressing the underlying cause of the deficiency.*

**Evaluation of the editorial functions of ChatGPT. Prompts are indicated, followed by the responses by GPT-4 or GPT-3.5.**

**Format compliance and language editing**

- *(Prompt) Can ChatGPT ensure format compliance with journal guidelines?*
*(GPT-4) ChatGPT can help you adhere to journal guidelines by providing suggestions and guidance, but it cannot guarantee perfect compliance as it requires your active involvement in editing and formatting the content.*
- *(Prompt) What type of language editing can ChatGPT perform?*
*(GPT-4) ChatGPT can perform various language editing tasks, including grammar and punctuation correction, sentence rephrasing, and content summarization.*
- *(Prompt) Shorten the text length of this abstract from 180 words to 50 words: (the abstract of this article is copied and pasted here).*
*(GPT-4) This study evaluates ChatGPT's potential in scientific writing using vitamin B12 as a case study, comparing GPT-4 and GPT-3.5. GPT-4 shows promise in streamlining scientific writing tasks but has limitations like bias, outdated information, and inaccuracies. While helpful, it cannot replace a scientist's expertise. Future versions may overcome current limitations.*
*(GPT-3.5) ChatGPT's ability to assist in scientific writing was evaluated using vitamin B12 as a case study. Results show potential for streamlining language editing and generating lists. Limitations include bias, lack of transparency, and inaccurate information, but accuracy of GPT-4 is superior to GPT-3.5. ChatGPT can be a helpful assistant but not a replacement for a scientist's expertise.*
- *(Prompt) Write this citation in The Lancet Digital Health journal style: Reynolds, Edward. “Vitamin B12, folic acid, and the nervous system.” The Lancet. Neurology vol. 5,11 (2006): 949-60. doi:10.1016/S1474-4422(06)70598-1*
*(GPT-4) Reynolds E. Vitamin B12, folic acid, and the nervous system. Lancet Neurol. 2006;5(11):949-60. doi:10.1016/S1474-4422(06)70598-1.*
*(GPT-3.5) Reynolds E. Vitamin B12, folic acid, and the nervous system. Lancet Neurol. 2006 Nov;5(11):949-60. doi: 10.1016/S1474-4422(06)70598-1.*
- *(Prompt) Rewrite this text: vitamen b12 tretemnt defecenci orl suplemnt jections hi*
*(GPT-4) Treatment for vitamin B12 deficiency includes oral supplements or injections.*

**Generating tables and figures**

- *(Prompt) Is it possible for ChatGPT to generate images or tables to represent data?*
*(GPT-4) ChatGPT cannot generate images or tables directly but can describe data representations that can be manually converted into visual formats.*
- *(Prompt) List the two most common natural vitamin B12 forms and their chemical formulas in table format.*
*(GPT-4)* 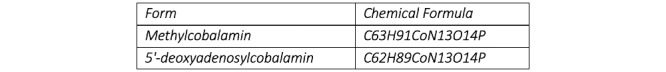

**Plagiarism check**

- *(Prompt) Can ChatGPT check for plagiarism?*
*(GPT-4) ChatGPT cannot directly check for plagiarism, but you can use specialized plagiarism detection tools like Turnitin or Grammarly for this purpose.*
- *(Prompt) Check this sentence for plagiarism: During pregnancy the absorption of B12 ensures the B12 status not only for the mother but also for the fetus.*
*(GPT-4) The given sentence does not appear to be a direct copy from a specific source, but it conveys a common idea about the importance of B12 absorption during pregnancy for both the mother and the fetus.*

**Keyword and abbreviation list**

- *(Prompt) Is it possible for ChatGPT to generate a keyword and abbreviation list for a text?*
*(GPT-4) Yes, ChatGPT can generate a keyword and abbreviation list for a text by extracting and summarizing key terms and their corresponding abbreviations.*
- *(Prompt) Create a five keyword list for a scientific article based on this abstract: (the abstract of this article copied and pasted here).*
*(GPT-4) ChatGPT in scientific writing, Vitamin B12 case study, GPT-4 vs GPT-3.5 comparison, Limitations and potential improvements, Responsible use and researcher caution*
*(GPT-3.5) ChatGPT, Vitamin B12, Natural Language Processing, Artificial Intelligence, Scientific Writing*
- *(Prompt) Create an abbreviation list for this article (the whole text of this article copied and pasted here).*
*(GPT-4) OpenAI - Open Artificial Intelligence, ChatGPT - Chat Generative Pre-trained Transformer, GPT-4 - Generative Pre-trained Transformer 4, GPT-3.5 - Generative Pre-trained Transformer 3.5, B12 - Vitamin B12*

**Abstract generation and data representation**

- *(Prompt) Can ChatGPT generate a title, abstract, and conclusion based on the results section text?*
*(GPT-4) Yes, ChatGPT can generate a title, abstract, and conclusion based on the results section text, provided it is given sufficient context and information about the topic.*
- *(Prompt) Write a scientific journal abstract and title based on this section (answer in 50 word): (the human written part of the results and discussion section of this article, till this prompt, copied and pasted here).*
*(GPT-4) Title: Evaluating the Scientific Text Generation and Editorial Capabilities of ChatGPT in the Context of Vitamin B12 Research*
*Abstract: This study assesses the scientific text generation and editorial functions of ChatGPT, specifically regarding vitamin B12-related content. Results indicate that while ChatGPT can generate coherent and informative answers, it may contain inaccuracies and biases. Furthermore, its editorial capabilities are promising, yet require careful editing by researchers. Users should critically evaluate ChatGPT-generated text and exercise caution when using it for scientific writing.*

## Discussion

### Principal Findings

The results suggest that GPT-4 can enhance scientific writing by aiding in language editing, abstract generation, and compiling keyword and abbreviation lists. Despite its strengths, it has notable limitations, including bias, outdated information, opacity, and inaccuracies. Additionally, it cannot perform plagiarism checks or provide accurate citations. Compared to GPT-3.5, GPT-4 delivers more precise responses.

Regarding the ability to generate text with proper references in the context of scientific writing, it is a major drawback that ChatGPT does not include the most recent knowledge. The answer to the question “What is vitamin B12?” seemed adequate from both models [21] and could be used in a research paper. The citations seemed acceptable; however, GPT-4 incorrectly included Allen as an author of the paper [22], while GPT-3.5 was mistaken both concerning the publication year and digital object identifier (DOI) [23]. Besides the example of incorrect references shown, a long list of references that do not exist or contain incorrect reference links or incorrect PubMed ID numbers was encountered (data not shown). For these reasons, asking for references was excluded in the rest of the prompts. More generally speaking, it is advised to avoid using current tool versions to identify references.

Overall, GPT-4 presented superior text for all 3 prompts assessing text accuracy and self-contradiction compared to GPT-3.5. GPT-4’s response to “who needs treatment” correctly stated that treatment was for individuals with vitamin B12 deficiency; however, the phrase “they may” at the end was inappropriate, as all vitamin B12-deficient individuals require treatment. GPT-3.5’s response to the same question mentioned “vitamin B12 deficiency” as one of various causes, rather than encompassing all causes. The answers to “How to treat pernicious anemia” were correct and similar for both versions of GPT but lacked details. Patients suffering from this disease will initially need several injections of B12 given within an interval of a few days. Maintenance therapy involves either injections with longer intervals or a daily high dose of oral vitamin B12 [24]. GPT-4’s response about intrinsic factor was accurate. In contrast, the answer by GPT-3.5 had serious flaws. Injection of intrinsic factor would be potentially dangerous and lack any treatment effect as the function of the protein is in the gastrointestinal tract [25]. The above examples highlight the importance of critically evaluating ChatGPT-generated text and not accepting it at face value, while also demonstrating the improvement in accuracy from GPT-3.5 to GPT-4.

For the bias and prioritizing prompts, the assertion that cyanocobalamin is the most commonly used pharmaceutical form of vitamin B12 is true [26]. However, the answers were incomplete for both GPT-4 and GPT-3.5. The commonly used form, hydroxocobalamin, was not mentioned. In addition, the assertion that methylcobalamin and adenosylcobalamin are generally more bioavailable and effective remains a subject of debate [26]. The bias of GPT-3.5 toward cyanocobalamin could be due to its extensive mention in the literature relative to other therapeutically used forms of vitamin B12. A PubMed search revealed 21 to 28 times more results for cyanocobalamin than hydroxocobalamin, adenosylcobalamin, or methylcobalamin. The prompt thus confirms the statement that responses given by ChatGPT may well be biased and, once more, underscores the need for critical review of scientific text generated by ChatGPT.

In regard to repeatability and reproducibility prompts, GPT-4’s responses were consistent and accurate, linking treatment to the cause and severity [24]. GPT-3.5’s responses were limited to maintenance treatment for a deficiency resulting from poor vitamin absorption. For vitamin B12 deficiency due to inadequate dietary intake (eg, in those following a vegan diet), injections are necessary only at the beginning, followed by daily vitamin pills containing a physiological dose for maintenance treatment [24]. Thus, once again, the answer provided by GPT-4 was superior to that provided by GPT 3.5.

For format compliance and language editing prompts, 2 common formatting requirements were tested: text length and citation style. The condensed abstract produced by GPT-4 successfully communicated the primary ideas of the original abstract, retaining its logical structure without introducing new details or altering its initial meaning. The response by GPT-4 adhered to the 50-word limit, using 50 words and presenting a smoother transition between covered points, while the GPT-3.5 version had a rougher flow and did not comply with the specified word limit. ChatGPT detected the desired citation style as Vancouver and partially conformed to its rules, accurately presenting the data sequence, author names, and punctuation. Nonetheless, GPT-3.5 incorrectly formatted the year by adding the month, and both versions did not use the required italics or boldface. Consequently, it is inadvisable to rely on ChatGPT for editing citation styles in its present condition. The language editing capabilities of ChatGPT are remarkable as it corrected and proposed superior alternatives for a sentence containing misspelled words, incorrect grammar, irrelevant terms, and poor structure.

As stated by ChatGPT, the tool cannot generate images. Regarding tables, although the tool indicated that it cannot generate them, it is capable of generating tables and editing them by adding or removing columns and rows, as well as modifying the text within the tables (data not shown). Of note is that the listed chemical formula of adenosylcobalamin was wrong [27]; the listed formula was of cyanocobalamin.

ChatGPT made it clear that it is not capable of conducting plagiarism checks. Despite this, when asked to assess a text for plagiarism, it yielded inaccurate outcomes instead of notifying the user about its limitations. Take, for example, a scenario where a sentence was extracted verbatim from the introductory section of a paper published in an open-access journal [28] and presented to ChatGPT for plagiarism evaluation. The response from ChatGPT incorrectly indicated that the sentence did not seem to be directly lifted from an identifiable source.

Interestingly, the list of keywords generated by GPT-4 was wordy and inappropriate, while that generated by GPT-3.5 provided a mostly relevant keyword list. The abbreviation list for the abstract was accurate, covering all abbreviations present. Generally, abbreviation lists should contain only pertinent abbreviations, but ChatGPT included all of them. For example, when asked to generate an abbreviation list for this entire article, it included “Ref: Reference” and “PubMed: U.S. National Library of Medicine's database of scientific research articles.” Although keyword and abbreviation lists produced by ChatGPT can be useful, they necessitate meticulous editing by the researcher.

When asked to compose an abstract based on the entire results and discussion sections, ChatGPT responded with:

The message you submitted was too long, please reload the conversation and submit something shorter.

Consequently, the prompt was shortened to include only the human-written discussion where the results were evaluated. ChatGPT’s capability to create a title and abstract based on this paper’s discussion section was impressive, though not perfect. The generated abstract was specific and included relevant conclusions, but it omitted any reference to the comparison between ChatGPT versions. Additionally, ChatGPT did not adhere to the 50-word limit specified in the prompt, using 62 words instead.

This study’s examination of ChatGPT’s capabilities was confined to the medical vitamin B12 domain, potentially limiting its findings and rendering them not fully transferable to other scientific areas; therefore, the findings may not reflect the model’s performance across varied fields of study. Despite attempts to ensure consistency by initiating each prompt on a new ChatGPT page, the intrinsic variability of AI response generation might still have posed challenges to the repeatability and reliability of the results. Additionally, the dependence on an external platform for accessing ChatGPT introduced potential vulnerabilities related to the models’ availability, stability, and timeliness of updates, which could have affected the study’s outcomes over its duration.

### Conclusion

Through prompts related to vitamin B12, potential was found in the GPT-4 model of ChatGPT for scientific writing. This tool could particularly benefit non-English speaking scientists by helping them streamline the language in their publications. This study revealed critical limitations of ChatGPT, mainly in relation to accuracy, bias, and references. The shortcomings warrant judicious use and avoiding its use as a substitute for human expertise. GPT-4 showed improvements compared to GPT-3.5, indicating promise for the further development of the tool. Overall, ChatGPT has the potential to be a useful tool for scientific writing, but researchers must be aware of its limitations and use it appropriately.

## Abbreviations

AI: artificial intelligence
DOI: digital object identifier

## Data Availability

The responses provided by ChatGPT used in this research can be accessed via the history feature on the ChatGPT website upon request. Similar outputs can be anticipated when identical input prompts as those used in this study are deployed by any user of the publicly available ChatGPT.
